# Carbon dots derived from *Zingiber officinale* Rosc (ginger) with hemostatic effects

**DOI:** 10.3389/fmolb.2025.1530469

**Published:** 2025-03-04

**Authors:** Wen-Jing Hu, Ai-Qi Yu, Hai-Zheng Bi, Zhao-Jiong Zhang, Zhi-Bin Wang, Meng Wang, Hai-Xue Kuang

**Affiliations:** Key Laboratory of Basic and Application Research of Beiyao (Heilongjiang University of Chinese Medicine), Ministry of Education, Heilongjiang University of Chinese Medicine, Harbin, China

**Keywords:** hemostatic effects, carbon dots, *Zingiber officinale* Rosc, carbonized ginger, traditional Chinese medicine

## Abstract

**Introduction:**

Ginger, as a traditional Chinese medicine (TCM), can be used in clinical practice to treat various diseases. The product of ginger processed at high temperatures is called carbonized ginger (CG), which has a hemostatic effect that ginger originally did not have. The purpose of this study is to investigate the hemostatic effect of CG and the substances that exert hemostatic effects.

**Methods:**

CG was prepared and successfully obtained CG carbon dots (CG-CDs) from its aqueous solution. After fully characterizing its structural information, the hemostatic effect was evaluated using mouse tail bleeding and liver injury bleeding models, and the clotting time was evaluated using capillary coagulation experiments. In addition, the hemostatic mechanism of CG-CDs was explored.

**Results:**

The average particle size of CG-CDs was observed to be 4.07 nm and the lattice spacing was 0.216 nm. It was mainly composed of graphite structured carbon, with the main constituent elements being C, N, and O, containing functional groups such as C=N, C=O, and C-OH. The FL spectrum showed that the maximum excitation wavelength of CG-CDs was 360 nm, and the maximum emission wavelength was 470 nm. The QY of CG-CDs was calculated to be 0.45%. CG-CDs shortened bleeding time, reduced bleeding volume, and also shortened the time for blood clotting. With the increase of CG-CDs, the values of FIB gradually increased, and the PT values gradually decreased. In addition, CG-CDs increased PLT count, increased PLT activating factor TXB2, decreased 6-keto-PGF_1*α*_, increased PAI-1, and decreased t-PA.

**Conclusion:**

CG-CDs obtained from CG has hemostatic activity, mainly by activating exogenous coagulation and co-coagulation pathways, increasing PLT count, increasing PLT activating factor TXB2, reducing 6-keto-PGF_1*α*_, increasing PAI-1, and reducing t-PA, thereby affecting the fibrinolytic system and other pathways to exert hemostatic effects.

## 1 Introduction

*Zingiber officinale* Rosc. (ginger) is an herbaceous plant in the Ginger family (Chinese Pharmacopoeia Commission, 2020). It is an important seasoning used to enhance the flavor of food or eliminate the fishy smell of meat and is also a traditional Chinese medicine (TCM) (Garza-Cadena et al., 2023). Its rhizome is often used for medicinal purposes and is recorded in the Chinese Pharmacopoeia (2020 edition). Ginger can treat symptoms such as bloating, abdominal pain, diarrhea, and vomiting caused by excessive consumption of cold foods. In addition, it can also relieve drug poisoning such as *Pinelliae* rhizoma and *Arisaematis* rhizoma, as well as food poisoning caused by excessive consumption of fish and crabs (Shaukat et al., 2023; Sulejmanović et al., 2024).

The use of carbonized TCM to treat diseases has a history of about 2,000 years. After a long period of clinical practice, the theory that carbonized TCM can be used for hemostasis has been formed. Ginger is processed at high temperatures to form carbonized TCM, also known as carbonized ginger (CG). Its hemostatic effect has been recorded in Chinese medical books for over a thousand years. It can be used for clinical treatment of various bleeding disorders, including rectal bleeding and vomiting bleeding (Zhang et al., 2024a). The difference in clinical effects before and after processing has aroused the exploration interest of many researchers. Upon reviewing existing literature, it was found that the material basis of the hemostatic effect of CG is mainly elucidated from the perspective of small molecule active compounds such as carbon, tannins, inorganic elements, and flavonoids (Yu et al., 2015). Despite years of effort, researchers have yet to reach a consensus, which greatly limits the clinical application of CG and hinders its further research and scientific applications. Therefore, there is an urgent need to explore the material basis directly related to the hemostatic effect of CG.

Carbon dots (CDs) are zero-dimensional nanomaterials that have attracted increasing attention from researchers due to their excellent aqueous solubility, biocompatibility, unique photoluminescence, and low toxicity (Hu et al., 2024). Since its discovery in 2004, it has rapidly become a new family of carbon-based nanomaterials that can be widely used in various fields such as biological imaging, ion detection, drug delivery, nanoprobes, tumor diagnosis and treatment (Xiao et al., 2024; Zhang et al., 2024b). There are two methods for preparing CDs: bottom-up and top-down. The top-down approach refers to synthesizing CDs by physically or chemically crushing carbon skeletons. The bottom-up approach utilizes organic molecules as precursors to polymerize CDs. The high-temperature pyrolysis method in the bottom-up approach is like the high-temperature processing method of TCM (Zhao et al., 2020a). This provides a new perspective and approach for studying the hemostatic properties of CG. Previous experiments have shown that CDs can be obtained from carbonized TCM, and multiple pharmacological experiments have shown that they have hemostatic effects. In addition, these compounds have the advantages of natural sources, safety and reliability, small particle size, and rapid hemostasis (Chen et al., 2019; Li et al., 2022). It is speculated that CDs may be the material basis for the hemostatic effect of carbonized TCM. This article provides a detailed exploration of the hemostatic effect of CG for the first time, providing a scientific basis for its hemostatic effect. This article uses the method in the Chinese Pharmacopoeia (2020 edition) to prepare CG, and then extracts, separates, and purifies its aqueous decoction, successfully obtaining CG-CDs. After fully characterizing its structural information, the hemostatic effect was evaluated using mouse tail bleeding and liver injury bleeding models, and the coagulation time was evaluated using capillary coagulation experiments. The hemostatic mechanism of CG was preliminarily explored, providing reference for its clinical application and the development of hemostatic new drugs.

## 2 Experimental

### 2.1 Plant materials and chemicals

Ginger (Batch number:20230501) produced in Sichuan Province, processed by Harbin Yutai Pharmaceutical Co., LTD., (Harbin, China). Dialysis membrane of 1,000-Da molecular weight cut-off (MWCO) was purchased from Beijing Biotopped Technology Co., Ltd. (Beijing, China). Hemocoagulase (HC) for injection was purchased from Jinzhou Ahon Pharmaceutical Co., Ltd., (Liaoning, China). All experiments were performed using deionized water (DW). Rat Enzyme-Linked Immunosorbent Assay (ELISA) kits for 6-keto-PGF_l*α*_, PAI-1,TXB_2_, and t-PA were purchased from Jiangsu Meimian Industrial Co., Ltd., (Jiangsu, China). Zingerone (Standard agent, Z09A10C94881) and 6-gingerol (Standard agent, M21HB178860) was purchased from Shanghai Yuanye Bio-Technology Co., Ltd. (Shanghai, China). Ginger (Standard agent, 120942-202112) was purchased from the National Institutes for Food and Drug Control. Pentobarbital sodium and other analytical-grade chemical reagents were obtained from Tianjin Fuyu Fine Chemical Co., Ltd. (Tianjin, China).

### 2.2 Animals

SPF-grade male Kunming mice (weighing 20.0 ± 2 g) and Sprague-Dawley (SD) rats (weighing 200 ± 20 g) were purchased from Changsheng Co., Ltd. (Liaoning, China, No. SCXK (LIAO) 2020-0001). All animals were raised in a controlled environment with a temperature maintained at around 25°C ± 5°C, humidity between 55% and 65%, and a 12 h light dark alternation cycle, with *ad libitum* access to food and water. The experiment was conducted strictly in accordance with the guidelines of the recommendations of National Institutes of Health Guide for Care and Use of Laboratory Animals and was approved by the Ethics Committee of Heilongjiang University of Chinese Medicine (No. 2023091404).

### 2.3 Preparation of CG-CDs

Firstly, 100 g ginger was processed into CG according to the method recorded in the Chinese Pharmacopoeia (2020 edition). If the surface is charred black, crispy, and the interior is brown, it is considered qualified. Weigh the prepared CG and calculate the yield using the following equation.

$$Y=M_{2}/M_{1}*100\%$$

Where Y (%) represents the yield of charcoal medicine, M_2_ (g) is the quality of medicinal materials after carbonization, M_1_ (g) is the quality of medicinal materials before carbonization.

**FIGURE 1:**
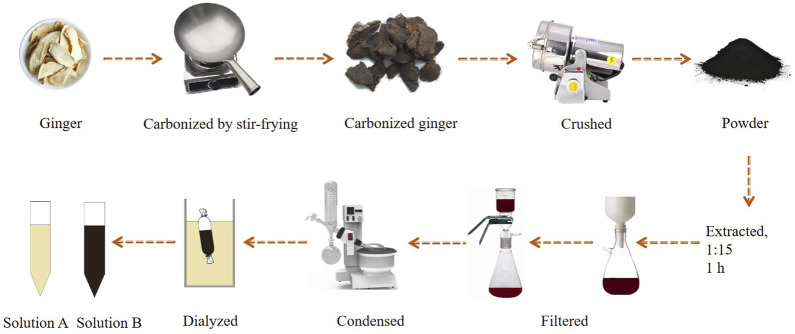
The entire preparation process for the CG-CDs.

CG was ground into fine powder, and then this powder was placed in a beaker and soaked in DW for 30 min. Then, the powder was boiled thrice at 100°C for 1 h each time. The resulting solution was filtered to remove residue. Next, the solution was concentrated using a rotary evaporator (N-1300; Tokyo Riken Instrument Co., Ltd., JPN), and purified using a dialysis bag. The entire preparation process for the CG-CDs is shown in Figure 1. The small molecules inside the dialysis bag were dialyzed out to obtain the solution outside the dialysis bag (solution A) and the CG-CDs solution (solution B). Finally, solution A and solution B need to be stored in an environment of 4°C for subsequent experimental operations.

### 2.4 Characterization of CG-CDs

Scanning Electron Microscope (SEM, Hitachi SU5000, JPN) was used to observe the surface morphology characteristics of ginger and CG. The size distribution and atomic lattice fringes of CG-CDs were observed by transmission electron microscope (TEM, Tecnai G2 F30, FEI, United States) and high-resolution transmission electron microscope (HR-TEM, Tecnai G2 F30, FEI, United States). Zeta potential and hydrodynamic size of particles in solution were measured on Zetasizer Nano ZS (Malvern Panalytical Ltd.). The CG-CDs were analyzed using a fast and non-damaging technique called powder X-ray diffraction (XRD, Bruker D8-Advance, Bruker, DE). The ultraviolet-visible (UV-vis) absorption spectra of CG-CDs were studied using a UV-vis spectrophotometer (UV-3600, Shimadzu, JPN) in a standard quartz cuvette. The surface functional groups of CG-CDs in the range of 400–4,000 cm^-1^ were studied using fourier transform infrared spectroscopy (FT-IR, 8400S, SHIMADZU, JPN). The samples were prepared by potassium bromide pellet method. Using dichloromethane: ethyl acetate: petroleum ether (60°C–90°C) (1: 1: 2) as the developing agent, the components in CG-CDs were identified by thin layer chromatography (TLC). The elemental composition and coordination of CG-CDs were detected and analyzed using X-ray photoelectron spectroscopy (XPS, ESCALAB 250XI, Thermo Scientific, United States). Fluorescence (FL, S1000/FS5, Edinburgh, United Kingdom) spectrometer was used to test the fluorescence properties and quantum yield (QY) of CG-CDs. The QY was determined with reference to quinine sulfate (% QY was 54 in 0.1 M sulphuric acid [H_2_SO_4_] solution) and calculated using the following equation.

$$Q_{\text{CDs}}=Q_{R}*\frac{I_{\text{CDs}}}{I_{R}}*\frac{A_{R}}{A_{\text{CDs}}}*\frac{\eta_{\text{CDs}}^{2}}{\eta_{R}^{2}}$$

where “Q” represents “QY”, “I” is the measured integrated area under the emission spectrum, “A” is the absorbance at 350 nm wavelength, and “η” is the refractive index of the solvent. “CDs” and “R” represent the CG-CDs and the standard, respectively.

### 2.5 Hemostasis studies of CG-CDs

The mouse tail amputation bleeding model and liver injury bleeding model were established according to previous reports (Zhao et al., 2020b). Briefly, animals adapt for a week. Then, 40 mice were randomly divided into 5 groups, with 8 mice in each group, namely, control group (normal saline [NS]), positive group (HC, 0.67 KU·kg^−1^), and the high-, medium-, and low-dose groups of CG-CDs (H: 0.078 g·mL^−1^, M: 0.156 g·mL^−1^, L: 0.321 g·mL^−1^). Except for the positive group, which was administered subcutaneously, all other groups were administered orally with a dosage of 0.2 mL. After 60 min of administration, the mice were anesthetized with pentobarbital sodium (50 mg·kg^−1^) via intraperitoneal injection.

In the tail amputation bleeding model, the mouse was placed on the operating table in a prone position and cut with surgical scissors at 1 cm from the tip of the mouse’s tail. Timing began when blood naturally flowed out, and blood was drawn from the tip of the tail every 30 s using filter paper until no more blood flowed out. Finally, weigh the filter paper with blood and record the results.

In the liver injury bleeding model, the mouse was incised along the midline of the abdomen to expose the liver using a sterile surgical knife while maintaining the mouse’s life. Then a small wound is formed on the liver, causing it to bleed. The bleeding was monitored and wiped with filter paper every 30 s until there was no more blood on the filter paper. Finally, weigh the filter paper with blood and record the results.

The haemostatic endpoint was defined as the maintenance of haemostasis for 30 min (Zhu et al., 2015). The bleeding time of the above two experiments was recorded until the haemorrhage ceased. After the experiment was completed, the mice were euthanized according to the guidelines of the Chinese National Standard “Guidelines for Euthanasia of Laboratory Animals” (GB/T 39760-2021) and the “AVMA Guidelines for the Euthanasia of Animals”.

### 2.6 Coagulation studies of CG-CDs

The capillary coagulation experiment was based on the methods described in the literature and made certain improvements (Han et al., 2023). Forty mice were randomly divided into five groups: normal group (normal saline [NS]), positive group (HC, 0.67 KU·kg^−1^), and high-, medium-, and low-dose groups of CG-CDs (H: 0.078 g·mL^−1^, M: 0.156 g·mL^−1^, L: 0.321 g·mL^−1^). The dosage and method of administration were consistent with the hemostasis experiment. These mice were anaesthetised with pentobarbital sodium (50 mg·kg^−1^) by intraperitoneal injection. One hour after the administration time, the mouse was cut at 1 cm from the tip of the tail, and the first drop of blood was discarded. Then, a capillary glass tube was used to collect blood from the tip of the mouse tail, and the entire capillary tube was filled with blood. Then start timing. Break a small section of the capillary tube every 30 s and slowly pull it open. The timing ends when filamentous substances appear in the blood inside the capillary glass tube, and this period is the clotting time. The experimental results were recorded.

### 2.7 Hemostatic mechanism of CG-CDs

This experiment referred to previous reports (Liang et al., 2023). Forty male SD rats were randomly divided into five groups (n = 8/group): normal group (normal saline [NS]), positive group (HC, 0.67 KU·kg^−1^), and high-, medium-, and low-dose groups of CG-CDs (H: 0.054 g·mL^−1^, M: 0.108 g·mL^−1^, L: 0.216 g·mL^−1^). The method of administration was consistent with the tail amputation bleeding experiment. One hour after administration, rats were anesthetized with pentobarbital sodium and blood was collected from the abdominal aorta.

Blood was collected and placed into EDTA anticoagulant blood collection tubes to detect PLT. Another blood sample was taken and placed in plastic tubes with 3.2% sodium citrate (citrate/blood: 1/9, v/v). The tube was left at room temperature for 1 h and then centrifuged at 3,500 rpm for 10 min. APTT, TT, PT and FIB were measured using an automatic coagulation analyzer (C2000-A, Mindray, China). The expression levels of 6-keto-PGF_1*α*_, TXB_2_, PAI-1, and t-PA in the plasma were evaluated using ELISA kits following the manufacturers. Blood samples were measured within 3 h. Store plasma at −80°C.

### 2.8 Statistical analysis

All data were processed using the statistical package for the social sciences (SPSS, version 25.0) for statistical analysis. The normally distributed data and homogeneous variances were expressed as the mean ± standard deviation . One-way analysis of variance (ANOVA) was used for statistical analysis of experimental data. The inter group differences were achieved through the least significant difference (LSD). *P* < 0.05 indicates differences in data statistics, while *P* < 0.01 indicates significant differences in data statistics.

## 3 Results

### 3.1 Characterization of CG-CDs

**FIGURE 2:**
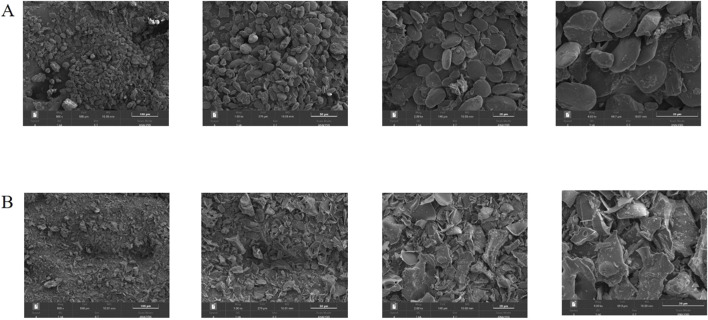
SEM images of ginger **(A)** and CG **(B)** at different magnifications (500 ×, 1,000 ×, 2000 ×, 4,000 ×).

After processing 100 g of ginger, 30.28 g of CG with a charred black and crispy surface and a brown interior was obtained. According to the formula, the carbonization yield was calculated to be 30.28%. The surface morphology characteristics of ginger and CG were measured using SEM as shown in Figure 2. Through observation, it was found that the surface morphology characteristics of ginger mainly appeared as round blocks, with slightly rough surfaces and occasional fragments. Moreover, the cross-section of the debris is irregular, accompanied by a large number of small debris particles, which can be seen at high magnification (4,000 ×). Therefore, it can be inferred that the structure of ginger has changed significantly after high temperature carbonization, resulting in many fragmented structures.

**FIGURE 3:**
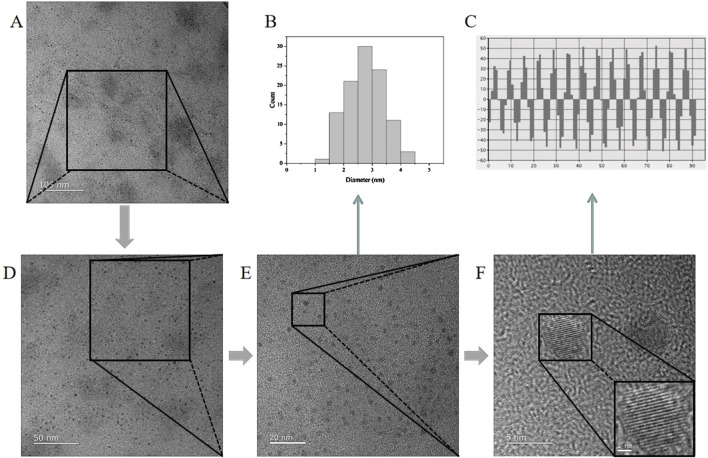
TEM and HR-TEM images of CG-CDs at different magnifications. **(A)** 100 nm, **(D)** 50 nm, **(E)** 20 nm, **(F)** 5 nm. **(B)** Histogram of particle size distribution of CG-CDs. **(C)** High resolution TEM image of atomic lattice fringes of CG-CDs.

**FIGURE 4:**
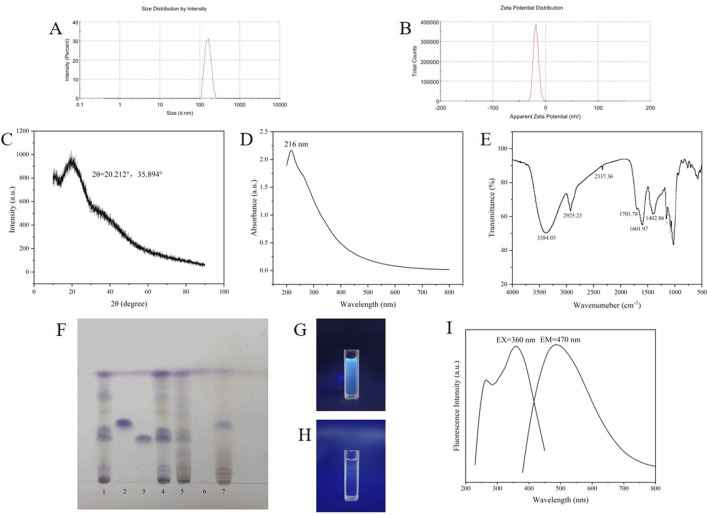
**(A)** CG-CDs particle size measured by DLS. **(B)** Zeta potential of the CG-CDs. **(C)** XRD pattern of CG-CDs. **(D)** UV-vis spectrum of CG-CDs. **(E)** FT-IR spectrum of CG-CDs. **(F)** Identification of CG-CDs (1. Ginger standard solution, 2. Zingerone standard solution, 3. 6-gingerol standard solution, 4. Ginger, 5. Ginger charcoal, 6. Solution B, 7. Solution A). **(G)** CG-CDs solution under 365 nm irradiation. **(H)** Aqueous solution under 365 nm irradiation. **(I)** Fluorescence spectrum of CG-CDs.

The TEM and HR-TEM results are shown in Figure 3, and the CG-CDs were distributed in a spherical shape with an average diameter of about 4.07 nm and a lattice spacing of 0.216 nm. The particle size of CG-CDs was evaluated using dynamic light scattering (DLS) technique. As shown in Figure 4A, the average particle size of CG-CDs is about 161 nm. The reason for the difference between DLS and TEM results may be that DLS measures the hydration diameter of CG-CDs in solution, while TEM directly measures the actual physical size of CG-CDs. Zeta potential analysis revealed that the potential of CG-CDs was −18.2 mV (Figure 4B).

Characterization of the crystal composition of CG-CDs by XRD (Figure 4C). CG-CDs had a strong diffraction peak and a weak absorption diffraction peak on the 2θ spectrum at approximately 20.212° and 35.894°. It is known that the 2θ in the XRD pattern of carbon in graphene is about 26°, and the reason for our deviation may be related to the highly disordered carbon atoms in the CG-CDs. It can be inferred that CG-CDs belong to an amorphous structure.

The UV-vis spectrum is shown in Figure 4D. The results have shown that CG-CDs have an adsorption peak at 216 nm. This may be due to the π-π* transition caused by the C=O bond (Guevara et al., 2024). Figure 4E shows the infrared spectrum of CG-CDs. There was a large and broad absorption peak at 3,384.03 cm^−1^, which was speculated to be the stretching vibration peak of the -OH bond. The peak at 2,925.23 cm^−1^ was an asymmetric stretching vibration absorption peak of the C-H bond in methylene CH_2_. The peak at 2,337.36 cm^−1^ was speculated to be the stretching vibration absorption peak of cumulative double bond conjugation, such as C=C=O, or the CO_2_ absorption peak in air. In addition, the absorption peak at 1701.78 cm^−1^ in the CG-CDs was the characteristic absorption of C=O bonds. The peak at 1,601.97 cm^−1^ may be attributed to the stretching vibration absorption peak of unsaturated C=C bonds. The characteristic absorption peak at 1,402.86 cm^−1^ was assigned as the bending vibration absorption peak of the saturated C-H bond, which occurred because of the methyl or methylene groups (Irshad et al., 2024).

As shown in Figure 4F, under sunlight conditions, ginger, CG and the standard solution showed clear spots of the same color at the corresponding positions on the thin layer plate, proving that ginger and CG met the pharmacopoeia standards. Solution B had no spots, while solution A had spots, indicating that most of the components of CG had been removed after dialysis. Additionally, solution B emits blue fluorescence under 365 nm ultraviolet light, while ordinary aqueous solution has no fluorescence (Figures 4G, H). The fluorescence characteristics of CG-CDs were analyzed. The maximum emission (EM) wavelength of CG-CDs was 470 nm, and the maximum excitation (EX) wavelength was 360 nm (Figure 4I). The QY of CG-CDs was calculated to be 0.45% using quinine sulphate as a reference.

**FIGURE 5:**
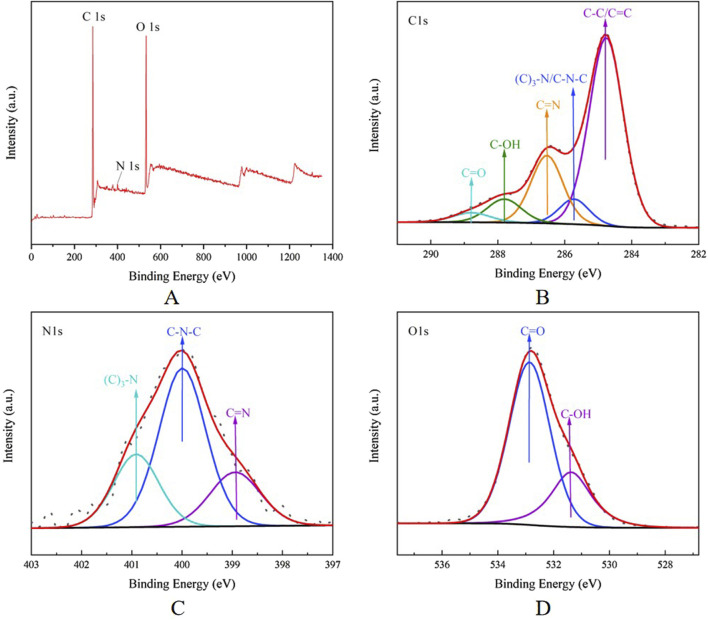
**(A)** Full survey spectrum XPS spectrum of CG-CDs. The high-resolution **(B)** C1s, **(C)** Ols, and **(D)** N1s XPS spectrum of CG-CDs.

According to the analysis results of XPS, the CG-CDs have obvious peaks at 284.8, 399.61, and 532.69 eV, indicating that the CDs were mainly composed of the elements C, O, and a small amount of N (Kasif et al., 2024). After analysis, it is known that the proportions of these elements were 75.34%, 22.59%, and 2.08%, respectively. In the high-resolution spectrum of CG-CDs, it can be seen that the C1s band can be resolved into five peaks at 284.76, 285.73, 286.53, 287.81, and 288.79 eV, which were attributed to C-C/C=C, (C)3-N/C-N-C, C=N, C-OH, and C=O, respectively. The Ols band can be resolved into 398.94, 399.99, and 400.91 eV. These peaks were corresponding to C=O and C-OH, respectively. The Nls band can be resolved into 531.37 and 532.86 eV, which were assigned to C-N-C, (C)_3_-N, and C=N, respectively (Figure 5).

### 3.2 Hemostasis studies of CG-CDs

This experiment investigated the hemostatic effect of CG-CDs in mice. The NS group had the longest bleeding time of 15.06 ± 1.88 min, while the HC group and the H, M, and L groups of CG-CDs (4.06 ± 1.21 min, 11.06 ± 1.50 min, 12.00 ± 1.46 min, 13.19 ± 1.89 min) all shortened the time for tail amputation in mice (Table 1). Compared with the NS group, there was a significant difference in bleeding time between the HC group and the H and M groups of CG-CDs (*P* < 0.05). There was no significant difference between the L group and the NS group (*P* > 0.05). From the results, it can be seen that the use of CG-CDs can significantly reduce the amount of bleeding in mice after tail amputation. As the dosage of CG-CDs increases, the amount of bleeding decreases accordingly. In addition, compared with the NS group, there was a significant difference in bleeding loss between the H and M groups of CG-CDs (*P* < 0.05).

**TABLE 1:** The effect of CG-CDs on mouse tail amputation bleeding model ( $\bar{\chi }$ ± S, n = 8).

Group	Total blood loss (g)	Time (min)
NS	0.2530 ± 0.0089	15.06 ± 1.88
HC	0.1249 ± 0.0058^**^	4.06 ± 1.21^**^
H	0.2001 ± 0.0090^**^	11.06 ± 1.50^**^
M	0.2382 ± 0.0106^*^	12.00 ± 1.46^*^
L	0.2405 ± 0.0116	13.19 ± 1.89

*Compared with the NS group, *P* < 0.05, and ** compared with the NS group, *P* < 0.01.

**FIGURE 6:**
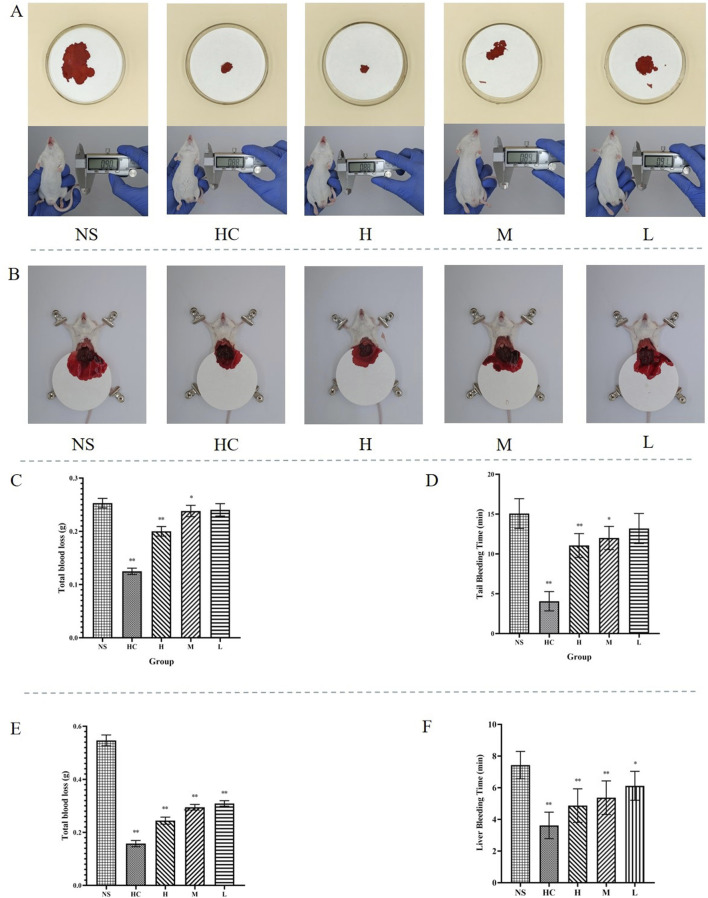
**(A)** Different treatments for hemostasis after bleeding in mouse tail amputation bleeding model. **(B)** Different treatments for hemostasis after bleeding in mouse liver injury bleeding models. **(C)** The total blood loss after different treatments in mouse tail amputation bleeding model. **(D)** The bleeding time after different treatments in mouse tail amputation bleeding model. **(E)** The total blood loss after different treatments in mouse liver injury bleeding model. **(F)** The bleeding time after different treatments in mouse liver injury bleeding model (^*^ *P* < 0.05; ^**^ *P* < 0.01, as compared with the NS group).

The mouse liver injury bleeding model was established to simulate organ bleeding and further evaluate the hemostatic ability of CG-CDs. As shown in Figure 6, compared with the NS group (7.44 ± 0.86 min), the three doses of CG-CDs, H (4.88 ± 1.06 min, *P* < 0.01), M (5.38 ± 1.06 min, *P* < 0.01), and L (6.13 ± 0.92 min, *P* < 0.05), can shorten the bleeding time of mouse liver, and the H group has the shortest hemostasis time. The experimental results showed that the NS group had the highest amount of bleeding, which was 0.5468 ± 0.0204 g. After administering CG-CDs, the amount of liver bleeding was significantly reduced (*P* < 0.01). The total blood loss of the H, M, and L groups of CG-CDs was 0.2442 ± 0.0133 g, 5.38 ± 1.06 g, and 6.13 ± 0.92 g, respectively (Table 2).

**TABLE 2:** The effect of CG-CDs on mouse liver injury bleeding model ( $\bar{\chi }$ ± S, n = 8).

Group	Total blood loss (g)	Time (min)
NS	0.5468 ± 0.0204	7.44 ± 0.86
HC	0.1577 ± 0.0118^**^	3.63 ± 0.83^**^
H	0.2442 ± 0.0133^**^	4.88 ± 1.06^**^
M	0.2948 ± 0.0109^**^	5.38 ± 1.06^**^
L	0.3093 ± 0.0102^**^	6.13 ± 0.92^*^

*Compared with the NS group, *P* < 0.05, and ** compared with the NS group, *P* < 0.01.

### 3.3 Coagulation studies of CG-CDs

**FIGURE 7:**
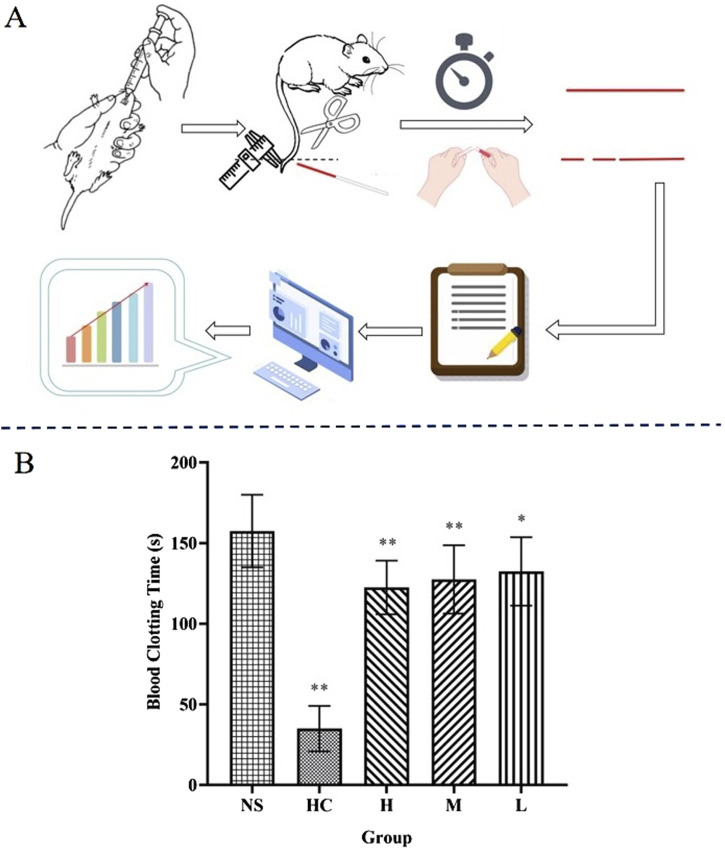
**(A)** Capillary coagulation experiment: Break a part every 30 s to observe the coagulation condition. **(B)** Coagulation time for different treatments after bleeding (^*^ *P* < 0.05; ^**^ *P* < 0.01, as compared with the NS group).

Blood coagulation is an essential part of the process of stopping bleeding (Zhang et al., 2024c). Compared with the NS group (157.50 ± 22.52 s), there were significant differences in coagulation time among all groups (*P* < 0.05), especially in the positive group (HC) and the H and M groups of CG-CDs (*P* < 0.01), and the L group (132.50 ± 21.21 s) had significant differences (*P* < 0.05) (Table 3) (Figure 7).

**TABLE 3:** The effect of CG-CDs on coagulation time in mice ( $\bar{\chi }$ ± S, n = 8).

Group	Dosage	Time (s)
NS	-	157.50 ± 22.52
HC	0.67 KU·kg^−1^	35.00 ± 14.14^**^
H	3.12 g·kg^−1^	122.50 ± 16.69^**^
M	1.56 g·kg^−1^	127.50 ± 21.21^**^
L	0.78 g·kg^−1^	132.50 ± 21.21^*^

*Compared with the NS group, *P* < 0.05, and ** compared with the NS group, *P* < 0.01.

### 3.4 Hemostatic mechanism of CG-CDs

**FIGURE 8:**
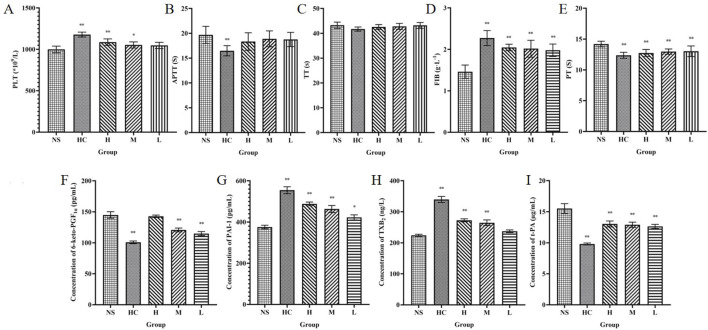
Haemostasis mechanism of CG-CDs. Platelets **(A)** activated partial thromboplastin time **(B)**, thrombin time **(C)**, fibrinogen **(D)** and prothrombin time **(E)**, 6-keto-prostaglandin F_lα_ **(F)**, plasminogen activator inhibitor-1 **(G)**, thromboxane B_2_ **(H)**, tissue plasminogen activator **(I)** were determined in the plasma of SD rats. (^*^ *P* < 0.05; ^**^ *P* < 0.01, as compared with the NS group).

The number of PLT is closely related to the hemostatic effect (Bian et al., 2024). The experimental results showed that compared with the NS group, both the HC group and the H and M groups of CG-CDs increased the number of PLTs (*P* < 0.05, *P* < 0.01). The L group of CG-CDs also increased the number of PLTs, but the difference was not significant (*P* > 0.05) (Table 4) (Figure 8).

**TABLE 4:** The effect of CG-CDs on PLT count and coagulation parameters in rats ( $\bar{\chi }$ ± S, n = 8).

Group	PLT (×10^9^/L)	APTT (s)	TT (s)	FIB (g·L^−1^)	PT (s)
NS	999.63 ± 40.48	19.68 ± 1.74	43.27 ± 1.27	1.46 ± 0.16	14.19 ± 0.46
HC	1,179.00 ± 29.52^**^	16.49 ± 1.03^**^	41.71 ± 0.83	2.27 ± 0.18^**^	12.37 ± 0.49^**^
H	1,087.88 ± 39.32^**^	18.34 ± 1.77	42.56 ± 0.97	2.05 ± 0.08^**^	12.74 ± 0.57^**^
M	1,053.63 ± 37.01^*^	18.89 ± 1.60	42.80 ± 1.21	2.02 ± 0.20^**^	12.97 ± 0.43^**^
L	1,048.00 ± 37.86	18.77 ± 1.43	43.20 ± 1.17	1.98 ± 0.15^**^	13.03 ± 0.86^**^

*Compared with the NS group, *P* < 0.05, and ** compared with the NS group, *P* < 0.01.

Compared with the NS group, H, M, and L groups of CG-CDs can significantly affect FIB and PT values (*P* < 0.05). With the increase of CG-CDs, the values of FIB gradually increased (1.98 ± 0.15 g·L^−1^, 2.02 ± 0.20 g·L^−1^, 2.05 ± 0.08 g·L^−1^), and the PT values gradually decreased (13.03 ± 0.86 s, 12.97 ± 0.43 s, 12.74 ± 0.57 s). CG-CDs had no significant effect on TT and APTT values (*P* > 0.05), but there was a decreasing trend in TT and APTT values with increasing dosage of CG-CDs.

As shown in the figure, the HC group had the lowest content of 6-keto-PGF_1α_, followed by the L, M, and H group. Compared with the NS group (144.98 ± 5.26 pg·mL^−1^), there were statistically significant differences (*P* < 0.01) in the HC (100.77 ± 2.13 pg·mL^−1^), M (120.69 ± 3.07 pg·mL^-1^), and L group (114.89 ± 3.20 pg·mL^−1^). The differences between HC, H, M and NS group were statistically significant (*P* < 0.01). Except for the HC group, the highest TXB_2_ content in the H group was 272.57 ± 4.84 ng·L^−1^, and the TXB_2_ content also increased with the increase of CG-CDs dose (Table 5).

**TABLE 5:** The effect of CG-CDs on relevant indicators in rat plasma ( $\bar{\chi }$ ± S, n = 8).

Group	6-Keto-PGF_1α_ (pg·mL^−1^)	PAI-1 (pg·mL^−1^)	TXB_2_ (ng·L^−1^)	t-PA (µg·L^−1^)
NS	144.98 ± 5.26	375.79 ± 8.89	224.06 ± 3.61	15.51 ± 0.80
HC	100.77 ± 2.13^**^	554.51 ± 16.67^**^	339.78 ± 9.76^**^	9.81 ± 0.15^**^
H	142.79 ± 1.97	488.20 ± 8.86^**^	272.57 ± 4.84^**^	13.03 ± 0.48^**^
M	120.69 ± 3.07^**^	463.33 ± 17.00^**^	264.76 ± 9.04^**^	12.90 ± 0.42^**^
L	114.89 ± 3.20^**^	422.54 ± 12.46^*^	237.22 ± 4.35	12.64 ± 0.32^**^

*Compared with the NS group, *P* < 0.05, and ** compared with the NS group, *P* < 0.01.

Compared with the NS group, the PAI-1 content increased in the HC, H, M, and L group. The HC, H, and M group showed significant differences (*P* < 0.01), while the L group showed significant differences (*P* < 0.05). The t-PA content in the NS group was 15.51 ± 0.80 μg·L^−1^. Intraperitoneal injection of CG-CDs in the H (13.03 ± 0.48 μg·L^−1^), M (12.90 ± 0.42 μg·L^−1^), and L group (12.90 ± 0.42 μg·L^−1^) all reduced t-PA content. The HC group (9.81 ± 0.15 μg·L^−1^) also significantly reduced t-PA content, with a significant difference compared to the NS group (*P* < 0.01).

## 4 Discussion

In daily life, it is inevitable for people to experience bumps, injuries, and bleeding. For example, sudden trauma or surgical treatment in hospitals. Normally, minor injuries and bleeding will automatically stop in a short period of time, causing less damage to the body (Liu et al., 2024). When encountering severe bleeding injuries, the body’s own coagulation system may not be able to quickly and effectively control bleeding, and specific technical means are needed to quickly achieve hemostasis, thereby improving the patient’s survival rate and quality of life (Chen et al., 2024). Physical hemostasis methods such as bandage wrapping, gauze filling, and pressure hemostasis, as well as medication hemostasis methods such as using anti-fibrinolytic drugs to prevent blood clot breakdown and enhance wound closure, have been applied and developed, but they each have their own limitations. Physical hemostasis methods may increase the risk of infection, while medication hemostasis may lead to thrombosis and may cause other treatment-related side effects. Although current research shows that some natural materials are used for hemostasis, they still face challenges such as biological safety, hemostatic effectiveness, and practical feasibility that need to be overcome (Zhang et al., 2024d). Therefore, effective control of hemostasis has been a focus and hotspot of research in recent years.

This experiment successfully prepared CG-CDs using ginger as a carbon source. The physicochemical properties of CG-CDs were determined by TEM, HR-TEM, XRD, FT-IR, XPS and other techniques. The particle size and distribution directly affect the physical stability of the nano dispersion system. DLS shows that the particle size distribution of CG-CDs is about 161 nm, with a PDI value of 0.187, indicating that the particle size distribution of CG-CDs is relatively uniform. The potential of the nano dispersed system determines whether the particles in the liquid exist stably. The higher the zeta potential, the denser the surface charge of particles, and the greater the electrostatic repulsion between particles, thereby enhancing the stability of particles in the liquid (Arshad et al., 2024). The zeta potential value of −18.2 mV indicates that CG-CDs have good stability in biological systems. A negative zeta potential value indicates that the CDs surface carries a negative charge. CG-CDs may be negatively charged due to the presence of carbonyl or hydroxyl groups on their surface.

Three models were used to verify the hemostatic effect of CG-CDs and explore the material basis for its hemostatic effect. The mouse tail amputation bleeding model and liver injury bleeding model represent non-lethal and mild injuries, and therefore cannot be generalized to all forms of bleeding in other parts, but they can be compared with other hemostatic drugs in this way. Under normal circumstances, the maintenance of vascular wall integrity and blood patency in the body is closely related to physiological hemostasis and blood coagulation systems (Mruthunjaya and Torriero, 2024). Therefore, this study also used capillary coagulation experiments to detect the coagulation effect of CG-CDs. Bleeding time, bleeding volume, and clotting time are commonly used indicators for screening hemostatic drugs (Han et al., 2023). The bleeding time represents the time required from natural flow to natural cessation. The amount of bleeding refers to the weight of blood lost during this period. These two parameters can reflect the platelet count, quality, and hemostatic function of capillaries. Coagulation refers to the process in which factor XII is activated and triggers a series of coagulation factors to be activated when blood comes into contact with a negatively charged surface after leaving the body, resulting in the transformation of fibrinogen into fibrin. The results of this experiment showed that CG-CDs can shorten the bleeding time, reduce the amount of bleeding in mouse tail amputation bleeding experiments and liver injury bleeding experiments, and also shorten the time for blood to show stringing phenomenon. It can be inferred that CG can exert hemostatic effects, possibly because the CDs in it have the ability to promote hemostasis and coagulation.

APTT, PT, TT, and FIB are four commonly used indicators for evaluating the coagulation system, which can reflect the types and concentrations of proteins in plasma (Lei et al., 2023). APTT and PT are performance indicators of endogenous and exogenous coagulation pathways, respectively. Both FIB and TT can reflect the function of plasma fibrinogen, and the activity of co-coagulation pathway and promoting the conversion of FIB to fibrin in plasma is related to the levels of TT and FIB (Wang et al., 2019a). Compared with the NS group, as the dose of CG-CDs increased, the FIB value gradually increased (*P* < 0.01) and the PT value gradually decreased (*P* < 0.01). However, CG-CDs had no significant effect on the values of TT and APTT (*P* > 0.05), but there was a decreasing trend in the values of TT and APTT with increasing dosage of CG-CDs. Therefore, it can be considered that the hemostatic effect of CG-CDs may be related to its promotion of the activation of exogenous coagulation pathways and co-coagulation pathways. And the increase in FIB value after treatment suggests that CG-CDs promote PLT aggregation and increase the content of FIB in the blood to promote thrombosis and achieve hemostatic effects.

PLT are a type of cell produced by megakaryocytes in bone marrow hematopoietic tissue, which lack nuclei and organelles (Mancuso and Santagostino, 2017). They are also an important component of human blood. The amount of PLT in normal blood will be maintained at a certain level. Some diseases may lead to a decrease or increase in the number of PLTs. PLT counting can determine whether the body tends to bleed and whether it has the ability to stop bleeding, which is also helpful for the diagnosis and differential diagnosis of hemostatic and thrombotic diseases in clinical practice (Picker S. M. 2011). This study found that CG-CDs have an impact on the quantity and activity of PLT. It is speculated that CG-CDs exert hemostatic effects by affecting the number and activity of PLT. After administration, the number and activity of PLT increase, making it more likely to form blood clots and exert hemostatic effects.

**FIGURE 9:**
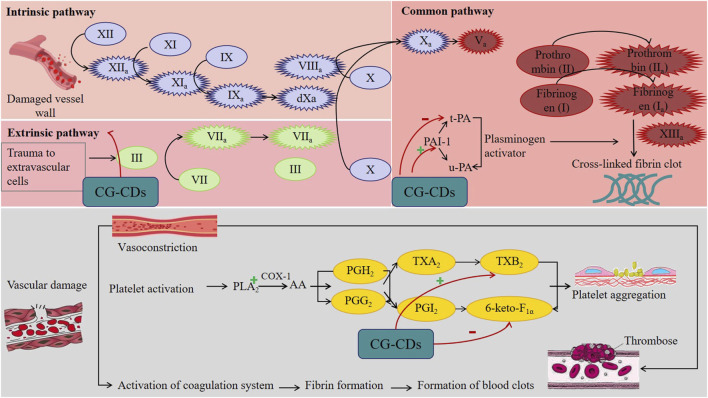
Potential mechanism of CG-CDs exerting hemostatic effect.

PGI_2_ and TXA_2_ exist in a certain proportion, and once the balance between the two is disrupted, it can cause PLT aggregation, thrombosis, and vasoconstriction in blood vessels. Based on the instability of both properties, their metabolites 6-keto-PGF_1α_ and TXB_2_ were selected as concentration detection indicators. The experimental results showed that CG-CDs can significantly increase the concentration of TXB_2_ and decrease the concentration of 6-keto-PGF_1α_ (Figure 9). A system composed of a series of chemical substances involved in the fibrinolytic process is called the fibrinolytic system. The fibrinolytic system mainly includes plasminogen, plasmin, plasminogen activator, and fibrinolytic inhibitors (Al-Ghafry et al., 2024). t-PA and PAI-1 are important physiologically active substances in the fibrinolytic system. The balance between the two is crucial for maintaining the normal function of the blood fibrinolytic system (Kramkowski et al., 2015; Morimoto-Kamata and Ohkura, 2024). Research has found that CDs can significantly increase the concentration of PAI-1 and decrease the concentration of t-PA, thereby affecting the hemostatic effect of the fibrinolytic system (Figure 9). This indicates that the hemostatic effect of CG-CDs is related to enhancing the activity of the fibrinolytic system. This study provides new insights into potential biomedical and healthcare applications in the field of bleeding control and lays a solid foundation for future drug discovery.

CDs derived from TCM usually have good biocompatibility. For example, some studies have validated the low toxicity of CDs to cells through cell experiments, with negligible cytotoxicity at lower concentrations and lower cytotoxicity at higher concentrations (Wang et al., 2019b; Liang et al., 2023). It can be inferred that CG-CDs also have good biocompatibility. Research has shown that nanoparticles with a diameter less than 100 nm can quickly come into contact with cells and be taken up (Gan et al., 2012). This is crucial for hemostatic drugs to quickly reach the bleeding site and take effect. Moreover, smaller nanoparticles have a larger specific surface area and can have more contact with coagulation factor VIIa, thereby affecting coagulation (Hao et al., 2019). In addition, charged nanoparticles can generate electrostatic interactions with blood cells or FIB with opposite charges, neutralizing surface charges and inducing their aggregation, promoting blood coagulation (Deng et al., 2011). It is speculated that the surface functional groups and negative charges exhibited by CG-CDs may be the main reasons for their hemostatic effect. Additionally, there are research reports that the precursor directly or indirectly determines the structure and function of CDs. CDs can inherit specific functions of precursors, such as reducibility, photosensitivity, and metal chelation. As a precursor of CG-CDs, CG has hemostatic properties, it is speculated that the hemostatic effect of CG-CDs also inherits from the precursor and combines the characteristics of CDs themselves, including small size, rich functional groups, and good biocompatibility. However, due to limited research and supporting data, more research is needed.

CG-CDs and CDs from other sources such as carbonized *Platycladus orientalis*, egg yolk oil, and carbonized *Schizonepetae Herba* all have good hemostatic effects. Revealing the differences in hemostatic effects and mechanisms of different CDs is of great significance for clinical practice. However, there is currently insufficient research in this area. *Scutellariae Radix Carbonisata* (SRC) has the effect of cooling blood and hemostatic. According to reports, SRC-derived CDs (SRC-CDs) have a protective effect on blood-heat and hemorrhage rats. It exerts anti-inflammatory and hemostatic effects by inhibiting the myD88/NF-κB signaling pathway, activating the fibrin system and endogenous coagulation pathways (Zhang et al., 2023). CG, as a carbon source for CG-CDs, has the effect of warming meridians and stopping bleeding (Zhang et al., 2024a). Modern pharmacological studies have shown that CG has a protective effect on deficiency cold and hemorrhagic syndrome rats (Li et al., 2023). We speculate that CG-CDs may also inherit this effect, which may be the unique effect of CG-CDs in hemostasis. This speculation deserves further research and verification. With the continuous deepening of nanoscience research, CG-CDs unique properties will continue to be discovered, and its application fields will also continue to expand. In addition, CDs can be functionalized on the surface to serve as nanocarriers for drug delivery, or specific ligands or molecules can be introduced to make CDs more targeted. Current research shows that CDs can serve as nanocarriers for delivering antibiotics and chemotherapy drugs (Osorio et al., 2024; Zhang et al., 2024b). Drugs can bind to functional groups on the surface of CDs through covalent or non-covalent bonds and achieve drug delivery through CDs to exert pharmacological effects. Future research can select suitable targeting ligands to modify the surface of CDs, enabling them to specifically bind to cells or tissues at the wound site, thereby improving hemostatic efficacy.

## 5 Conclusion

CG-CDs were extracted and separated from carbonized ginger, and their components were analyzed, and their physicochemical properties were characterized using TLC and nanotechnology techniques. Pharmacological experiments have shown that CG-CDs have hemostatic activity, mainly by activating exogenous coagulation pathways and co-coagulation pathways, increasing platelet count, and increasing platelet activating factor TXB_2_, reducing 6-keto-PGF_1α_, increasing PAI-1, and reducing t-PA, thereby affecting the fibrinolytic system and other pathways to exert hemostatic effects. To our knowledge, this is the first study to demonstrate the hemostatic effect of CG-CDs, which provides a research basis for future studies on the hemostatic effect of ginger charcoal and also offers a new strategy for exploring charcoal medicine hemostasis.

## Data Availability

The original contributions presented in the study are included in the article/Supplementary Material, further inquiries can be directed to the corresponding author.

## Abbreviations

APTT, Activated partial thromboplastin time; DW, Deionized water; DLS, Dynamic light scattering; ELISA, Enzyme-Linked Immunosorbent Assay; FL, Fluorescence spectrophotometer; FT-IR, Fourier transform infrared spectrometer; FIB, Fibrinogen; HC, Hemocoagulase; HR-TEM, High resolution transmission electron microscope; 6-keto-PGF_lα_, 6-keto-prostaglandin F_lα_; PAI-1, Plasminogen activator inhibitor-1; PLT, Platelets; PT, Prothrombin time; QY, Quantun yield; SEM, Scanning electron micrograph; TCM, Traditional Chinese medicine; TEM, Transmission electron microscopy; TLC, Thin-layer chromatography; TT, Thrombin time; TXB_2_, Thromboxane B_2_; t-PA, Tissue plasminogen activator; UV-Vis, Ultraviolet-visible spectrophotometer; XPS, X-ray photoelectron spectroscopy; XRD, X-ray powder diffractometer.
