# Supplementary figures and images for: Carbon dots derived from Zingiber officinale Rosc (ginger) with hemostatic effects
Source: Front Mol Biosci. 2025 Mar 4;12:1530469. doi: 10.3389/fmolb.2025.1530469 (PMC11913708; doi:10.3389/fmolb.2025.1530469)

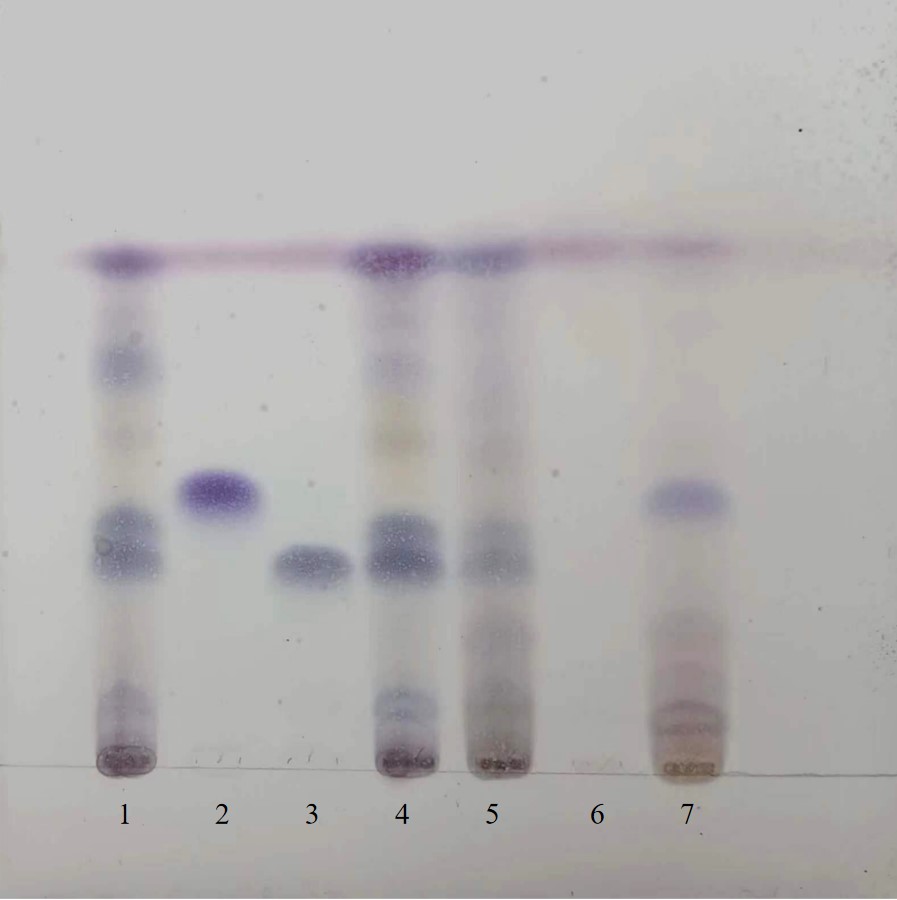

Supplement: Supplementary file 1 [file Image1.jpeg]
